# Indents

**Published:** 1916-08

**Authors:** 


### INDENTS

**☞ Brief Articles of Technical or Scientific Value will receive notice and comment. Contributions invited.**

*Dentist Honored.* Francis Mark Farmer, L.D.S., has received a Knighthood for rendering invaluable services to the English War Department. Sir Francis Farmer has his honor in particular for services as a dental surgeon. This is the third English dentist to receive such an honor, Sir John Tomes and Sir Edwin Saunders being the other members of the profession to be Knighted.

*Lack of Dentists.* Owing to the general enlistment of the younger dentists, the work of caring for the school clinics in England has been temporarily suspended.

*Exemption from Service of Dentists.* The Central Tribunal in England has ruled that, owing to the scarcity of registered dentists in England, dentists can claim exemption from conscription if they so desire. It would seem that as a measure of self-preservation some professional men should be left at home.

*Clean Modeling Compound from Trays.* After removing the excess or bulk of material saturate a pledget of cotton with chloroform and remove the adhering compound.—*Digest.*

*Amend English Dental Act.* At a recent session of the General Medical Council of England, a resolution was adopted to the effect that the act regulating license to practice dentistry should be amended. It was also thought wise to reform the curriculum of study. To what extent this is likely to be done was not suggested.

*To Avoid Bubbles in Casting.* Paint the investment over the surface with a brush made by winding a few shreds of cotton on a wooden toothpick, and apply the investment by dropping it on the surface of the wax model. Don't smear it on. It may be made to flow to place by blowing it with the chip blower.— M. J. Ruzicka, in *Digest*.

*The Pathological Significance of Impacted and Unerupted Teeth.* Lyons is of the opinion that the impacted tooth and the less common unerupted tooth, despite their often apparent harmlessness, may be causative factors in many nervous disturbances and other ills afflicting mankind. The teeth most commonly impacted are the lower third molars, the cuspids being close seconds. The most common sequela, especially with third molars, is local infection, which not infrequently involves the adjacent soft tissues including the fauces. Pressure resorbtion of second molars is another of the evils of impaction, as is encroachment upon the inferior dental canal. The author believes that the lower third molar is probably responsible for more reflected pains than is any other organ of the mouth or jaws. Apropos of reflex pains, he refers to the work of Goldscheider, who believes that the impulse of pain is conveyed to a nerve cell, with two nerve fibres leading from it, so that the brain may interpret the pain at the nerve ending of either nerve fibre. Examples of this are common in trifacial neuralgia where the source of irritation may be in one division of the trigeminus and the seat of pain in another. Reference is made to four cases of epilepsy which cleared up after removal of impacted third molars, and the belief expressed that impacted teeth should be considered as a possible causative factor in many of the obscure nervous diseases in which the trigeminus may possibly be involved. Regarding the unerupted tooth, the author states that the cause for such non-eruption is a pathological mystery. Attention is called to the fact that such teeth may remain in the jaw for years without giving trouble and suddenly become the seat of purulent inflammation. Also to the fact that the unerupted tooth is frequently associated with cystic growths, and that the vast majority of such growths occur in patients under thirty years of age, usually at the time when the affected tooth should erupt. All such teeth should be regarded with suspicion.—C. J. Lyons, abstracted by J. A. D. S. from *J. N. D. Assn.*

*Diagnosis.* First of all examine the gums; second, the teeth for masticating efficiency; third, for cavities; fourth, the mouth for tumors, new growths or other abnormalities. The abnormal color of the gum may lead us to a minute examination which may unearth hidden ledges of calculus, perhaps, on lingual surfaces of upper incisors, or unsuspected pockets, containing fermenting debris or pus; and further to a general physical examination of the patient—heart, lungs, urinary and salivary analyses, blood pressure, blood count, and bacteriological examinations of the secretions and debris round necks of teeth. To test masticatory efficiency I find it a *sine qua non* that models be made of the mouth so that the occlusion may be tested from the lingual aspect as well as the buccal and labial.—M. Quinby in *Journal A. S.*

*Dietetics and Dental Caries.* Of all national needs, a thorough investigation of dental caries and its primary and real (not its secondary and incidental) cause is most urgent. If we can spend millions to study occasional diseases such as infantile paralysis and pellagra, why not more to check this most common disease which is daily sapping the health and wealth of the land, and which, moreover, is indicative of some hidden unhygienic condition which produces degenerate human bodies? Recent investigations, which seem to lay the blame on faulty feeding in childhood, *i. e.*, administration of too much pap, at the expense of foods which require mastication, promise to lead to a real prophylaxis. This would be a notable achievement on the part of dietetics.—Editorial in *N. Y. Medical Journal.*

*Sciatica.* Besides removing the cause of sciatica, if such is possible, the general treatment is important. The diet should be modified according to the general condition. In some instances the meat should be removed from the diet. In other instances it should not be withdrawn; in fact, protein should be pushed and nutrition increased. Constipation should always be prevented. The treatment may be started with a brisk purge, as castor oil or calomel. The bowels should be moved daily, with salines if there is plethora, overweight and a good heart, and with vegetable laxatives if the patient is underweight, anemic, or with poor circulation.

Rest of this nerve is of primary importance, even if there is only a neuralgic condition, that is, an irritation without a neuritis or perineuritis, and it is imperative when there is a neuritis or perineuritis. A neurotic habit of the individual should always be noted. If such is present, this pain, like any other, will be harder to combat, and the main treatment should be toward the general condition, whether neurasthenic, hysteric or plainly neurotic. Like any other painful nerve, it must be kept warm. Warmth is one of the most important elements of local treatment.

In acute inflammations of this nerve the pain is so excessive that generally morphin must be given. But before morphin is resorted to, the leg should be immobilized and sometimes made rigid with a long splint. It is generally best not to give atropin with morphin, as atropin acts only on the peripheral endings of nerves, and in this case the nerve trunk is in trouble, and the atropin will combat somewhat the sedative action of the morphin. Also the morphin may have to be repeated more frequently than it would be wise to repeat the atropin. When the pain is not sufficiently severe to require morphin, antipyrin will sometimes act as a valuable sedative; also novaspirin, especially if the patient is rheumatic.

Besides the local treatment already suggested, dry cupping and cauterizing, and at times blistering along the course of the nerve, may be not only of temporary but also of permanent benefit. Sometimes the d'Arsonval high frequency current is of benefit in sub-acute and chronic cases. Sooner or later gentle massage is of benefit.

When this nerve is somewhat stretched and at rest, especially when the leg is surrounded by dry heat, as by hot sand bags or other methods of applying heat, excruciating pain may entirely cease. Such patients are fortunate, as narcotics need not be given.

If there is a rheumatic condition present, large doses of salicylates are of benefit. Alkalies are frequently of service, such as potassium citrate in 2-gram doses, in wintergreen water, given three or four times in twenty-four hours. If there is a nervous irritability and considerable cerebral irritation, bromids are of value, but they should be soon discontinued, as they interfere with nutrition and are very depressant, and calcium should be substituted.

Sometimes warmth is well applied to this nerve by a cradle containing electric lamps, and the leg is subjected to this kind of heat and the resulting local sweating once or twice a day for from fifteen minutes to half an hour.

High frequency currents have been applied to this painful nerve, sometimes with good results; but often electricity in any form is not well tolerated.

Ethyl chlorid spray along the course of the nerve or in different painful regions of the nerve has been reported to be successful in modifying the pain. Epinephrin ointments have been gently rubbed into the skin along the course of the nerve with some success.

Salt solutions, cocain solutions and beta-eucain solutions have been injected around the nerve sheath in prolonged acute and subacute cases with considerable success. The injection treatment with sterile water or sterile physiologic saline solution is an old treatment, and is recently being revived. This treatment is not advised in every case of sciatic pain, as many individuals are benefited and recover from ordinary local and constitutional treatment; but when the inflammation and pain persists, it is perhaps the most successful treatment.—*Journal of M. A.*, March 18, 1916.

*Dosage in Pressure Anesthesia with Cocain, and Danger Involved.*

The opinion is prevalent that pressure with cocain involves no danger whatever. The following experience, therefore, may serve as a warning: The writer was in the habit of using 1/6 grain tablets of cocain and adrenalin, placing them in position, moisting them with distilled water and applying pressure with a piece of gutta percha. This method was found very effective, and no hesitation was felt in using two tablets when required. On one occasion, however, when an upper left canine proved very obstinate, three tablets were employed. Still the pulp anesthesia was not complete, and while the pressure was continued, the patient began to show signs of cocain poisoning. The pupils of the eyes dilated enormously, the patient became pale and seemed not to understand when spoken to. She then became stiff, relapsing into coma, and becoming very limp. She was laid on a sofa and given 1/66 grain of strychnin by a physician, the dose being repeated, as the symptoms were not checked. The patient thereupon began to improve, but it was about four hours before she could be declared out of danger and the relapses into coma had ceased; and it was about another three hours before she was equal to being taken home in a cab. This case, of course, explains itself by the fact that a solution of probably from twenty to thirty per cent. of cocain was being injected into the patient through a very deeply implanted needle, as it were, *i. e.*, the apex of the tooth. Though a comparatively small proportion of the solution went through the apical foramen, it proved too much.—W. H. R. Grant, *Dental Cosmos.*

*Radiodermititis Following X-Ray Examination of the Teeth.* Recognizing the importance of the X-ray in dentistry, and the increasing number of dentists who are making use of it in their offices, MacKee urges that those who undertake to do X-ray work acquire the requisite knowledge and experience properly to qualify them for it; that in the X-ray we have a powerful and dangerous agent which is not fool proof, and, in order to avoid danger to himself and patient, the dentist should be thoroughly acquainted with its physics and biologic effect. A warning is raised against the installing of an outfit with no more knowledge of its potentialities than is required to start the apparatus, to actuate the tube, to place the tube, to produce the proper exposure and to develop the film. He proceeds by asking a list of questions which, he asserts, would probably prove embarrassing to the amateur radiologist in case of legal action against him. Attention is called to eight cases of radiodermatitis produced by X-ray examination of the teeth which have come under the observation of the author during the last sixteen months. Fortunately, these proved to be nothing more than an erythema lasting from three weeks to four months. In three cases the eye lashes, eyebrows and a portion of the scalp hair fell out, but returned in all but one case where the hair failed to return in the outer half of the eyebrow. Faulty technique was responsible in each case. Belief is expressed that the dentist may become expert in the production and interpretation of dental radiographs, providing he is willing to devote time and money to this work. Only such persons should make use of the X-ray.—Geo. M. Maskee in *Cosmos*.

*Facilities in Removing Teeth from a Rubber Plate.* Put the plate in boiling water, keep it there for five minutes while boiling. You will then find the rubber soft and easy to remove the teeth with any pointed tool. While secured from cracking, they are removed thoroughly clean from rubber.—*Brooklyn Dental Laboratory.*

*Dental Hygiene.* A monograph of the Department of Health of the city of New York states that it is safe to assume that not less than ninety per centum (832,500) of school children of the city are in need of dental treatment.

It may be said that this situation is due to imperfect tooth structure, the causes of which lie partly at least in prenatal malhygiene, improper feeding of children, lack of cleansing and neglect to prevent progressive decay by early professional treatment.

The insidious character of tooth decay and disease is becoming increasingly apparent from the scientific relationships being drawn between oral malhygiene and disease.

There are hardly enough licensed dentists to repair the dental ills of the school population alone. This situation, engaging the attention of school hygienists, has directed attention to new means of attacking this problem.

An advisory committee of dentists made the following report to the New York Health Commissioner:

''The sub-committee on dental hygienists held three meetings, at which the question of dental hygienists was thoroughly discussed. The committee now begs leave respectfully to report that a trial of surface cleansing of teeth of school children with accompanying instruction in oral hygiene be commenced at the earliest possible moment in one or more centers, preferably public schools, provided that the work be done by specially and adequately trained persons and under the supervision of competent directors."

On February 17, 1916, the whole Committee on Child Hygiene of the Advisory Council of New York ratified the report and recommendations of the sub-committee.

*Burnishing Silicate with Glass Rod.* When making a gold inlay for an anterior tooth and placing a silicate facing over the exposed portion it will be found advantageous to burnish the silicate to place with a round glass rod which has been bent in a Bunsen burner to a foot-plugger form. This works most perfectly for these combination fillings.—M. J. Homan, in *Dental Review.*

